# *bla*_IMP-4_-associated imipenem susceptibility discordance in the *Klebsiella pneumoniae* complex: characterization and a phenotype-guided confirmatory workflow

**DOI:** 10.1128/spectrum.01493-26

**Published:** 2026-10-14

**Authors:** Haoyun Lin, Danxuan Lin, Yanchen Li, Zihan Zhao, Jingsong Wu, Tingting Xu, Jiayu Wu, Jine Cheng, Xiaopeng Yuan

**Affiliations:** 1 Department of Laboratory Medicine, Shenzhen Key Laboratory of Pathogenic Microbiology and Antimicrobial Resistance Surveillance, Shenzhen People’s Hospital, The First Affiliated Hospital, Southern University of Science and Technology; The Second Clinical Medical College, Jinan University 255310, Shenzhen, China; 2 Guangdong Provincial Clinical Research Center for Laboratory Medicine, Guangzhou, China; 3 Guangdong Provincial Key Laboratory of Infection Immunity and Inflammation, Department of Pathogen Biology and Immunology, Shenzhen University Medical School, Shenzhen University 47890, Shenzhen, Guangdong, China; 4 School of Life Science, Bengbu Medical College 74539, Bengbu, Anhui, China; Tel Aviv Sourasky Medical Center, Tel Aviv, Israel

**Keywords:** *Klebsiella pneumoniae *complex, antimicrobial susceptibility testing, VITEK 2, broth microdilution, *bla*_IMP-4_, carbapenemase, imipenem, diagnostic workflow

## Abstract

Automated antimicrobial susceptibility testing (AST) may generate carbapenem minimum inhibitory concentration (MIC) results differing from reference methods, complicating recognition of carbapenemase-producing *Klebsiella pneumoniae* complex isolates. We characterized carbapenem susceptibility discordance in a retrospective discovery cohort of 133 carbapenemase-producing isolates collected in Shenzhen (2004–2017) by comparing imipenem (IPM) and meropenem (MEM) MICs obtained by VITEK 2 and broth microdilution (BMD). Confirmed discordance was defined as repeat-confirmed raw MIC differences of ≥2 log_2_ dilutions. Features were assessed by whole-genome sequencing, mCIM/eCIM, and exploratory phenotypic assays. Cross-card reproducibility was assessed using an alternative VITEK 2 AST card. A separate retrospectively enriched cohort of 166 *K. pneumoniae* isolates (2018–2024) was used to evaluate a phenotype-guided reflex-testing workflow. Twenty-nine isolates showed confirmed carbapenem AST discordance, including 28 with IPM discordance. Of these, 27 had raw VITEK 2 IPM MICs at least 2 log₂ dilutions higher than those obtained by BMD, whereas one was reported as resistant after system adjustment despite a low raw MIC. All discrepant isolates carried *bla*_IMP-4_ as the sole detected carbapenemase gene, had relatively low carbapenem MICs by BMD, and were distributed across 14 sequence types. Cross-card retesting reproduced the direction of IPM elevation, although the magnitude varied. In a combined internal analysis set of 275 isolates, the phenotype-guided workflow identified unflagged ≥2 log₂ MIC discrepancies with 81.4% sensitivity (95% CI, 67.4%–90.3%) and 92.2% specificity (95% CI, 88.1%–95.0%). These findings define a repeat-confirmed *bla*_IMP-4_-associated, IPM-predominant discordance between VITEK 2 and BMD and support phenotype-guided reflex-testing to identify unflagged MIC discrepancies and prioritize confirmatory testing in routine laboratories.

**IMPORTANCE:**

Clinical microbiology laboratories rely on automated systems to support antimicrobial susceptibility interpretation, but these systems may not always recognize method-dependent carbapenem susceptibility discordance. In this study, we show that a specific group of bacteria carrying the *bla*_IMP-4_ gene can produce potentially misleading carbapenem susceptibility results, particularly for imipenem, when tested by automated methods. These discrepancies may not be flagged by the instrument and could therefore be overlooked in routine practice. By combining routine laboratory data with genetic analysis and additional testing, we identify a recognizable pattern associated with these hidden discrepancies and examine a phenotype-guided confirmatory workflow to support their laboratory recognition. Our findings highlight the importance of targeted confirmatory testing and provide a framework for local validation of laboratory-actionable carbapenem susceptibility discrepancies, thereby supporting more reliable AST interpretation and antimicrobial resistance surveillance.

## INTRODUCTION

Carbapenem-resistant *Klebsiella pneumoniae* (CRKP) remains a major challenge for infection control and antimicrobial therapy in hospital settings (1–4). Its clinical management depends not only on the underlying resistance mechanism but also on how accurately the laboratory recognizes and interprets the corresponding resistance phenotype. Increasing evidence indicates that antimicrobial susceptibility testing (AST) results for carbapenemase-producing bacteria do not always align across testing methods (5–7). Such discordance may lead to two different consequences: potentially active agents may be unnecessarily excluded, whereas isolates carrying clinically relevant resistance mechanisms may be misinterpreted as having a different resistance phenotype. In addition to the biological complexity of the organism, method-dependent variation in laboratory testing therefore represents an important source of uncertainty for clinical decision-making and infection control.

Automated AST systems are routine tools in clinical microbiology laboratories, but interpretation becomes more challenging in carbapenemase-producing isolates (8, 9). This complexity reflects both the diversity of carbapenemase enzymes and bacterial backgrounds (10, 11), particularly in isolates showing low-level carbapenem resistance or carrying coexisting resistance determinants, which often result in ambiguous MICs (12, 13). Although low-level carbapenem phenotypes in carbapenemase producers and the limitations of automated AST systems are not new observations, their practical consequences for routine laboratory recognition, review, and confirmatory testing remain incompletely defined. Current screening frameworks, such as the EUCAST recommendation to investigate Enterobacterales with meropenem MICs > 0.125 mg/L or inhibition zone diameters < 28 mm for carbapenemase screening, underscore the diagnostic relevance of low-level carbapenem phenotypes (14); however, the operational consequences of applying such low-threshold screening across routine AST platforms and local epidemiologic settings remain insufficiently characterized.

Within this broader diagnostic context, certain carbapenemase genotypes may be especially prone to low-level or platform-dependent susceptibility discordance. Among them, *bla*_IMP-4_-positive *Klebsiella pneumoniae* complex isolates remain insufficiently characterized in routine AST interpretation. It is unclear whether these isolates display a reproducible, platform-dependent pattern of susceptibility discordance, particularly between automated AST and broth microdilution (BMD). It is also unclear whether they share accompanying phenotypic features beyond carbapenem MIC values that could assist recognition. More importantly, whether such features can be translated into a practical follow-up strategy for confirmatory testing has not been systematically evaluated. These questions are laboratory-relevant because such isolates may be phenotypically grouped with more strongly resistant organisms when automated systems report inflated MICs, or may escape further review when discrepant results are not explicitly flagged by the instrument.

Against this background, we characterized the pattern of carbapenem susceptibility discordance between VITEK 2 and BMD in a retrospective discovery cohort of carbapenemase-producing *Klebsiella pneumoniae* complex isolates, with particular attention to repeat-confirmed IPM-predominant discrepancies. We then assessed the associated genotype and phenotype features, including carbapenemase gene distribution, broader antimicrobial susceptibility profiles, and exploratory low-inoculum IPM–MEM colony-recovery patterns. Finally, we evaluated a phenotype-guided confirmatory workflow in a separate retrospectively enriched cohort to explore its potential utility for routine laboratory recognition of susceptibility discrepancies that were not flagged by the testing system.

## MATERIALS AND METHODS

### Study design and isolate collections

This study included two retrospective isolate collections. All isolates were non-duplicate clinical isolates. The discovery cohort comprised 133 carbapenemase-producing isolates recovered from clinical specimens in the Shenzhen region between 2004 and 2017. These isolates had been identified in routine laboratory practice as *K. pneumoniae* and were subsequently analyzed by whole-genome sequencing (WGS) for species-level assignment, multilocus sequence typing, and resistance gene characterization. This cohort was used to characterize carbapenem susceptibility discordance between VITEK 2 and BMD and to assess associated genotype and phenotype features.

A separate retrospectively enriched workflow-evaluation cohort of 166 non-duplicate *K. pneumoniae* isolates was assembled from a large tertiary hospital in Shenzhen between 2018 and 2024. These isolates were selected from routine clinical microbiology records on the basis of suspicious carbapenem susceptibility results, including IPM MICs of 2–8 mg/L, instrument outputs requiring further review, or unusual resistance patterns such as IPM MICs ≥ 16 mg/L with retained susceptibility to aztreonam and/or amikacin or trimethoprim-sulfamethoxazole. This cohort was intended as a proof-of-principle evaluation of the workflow rather than as a population-based or consecutive validation cohort.

### Phenotypic and genotypic characterization

Carbapenemase production was assessed phenotypically using the modified carbapenem inactivation method (mCIM) and EDTA-modified carbapenem inactivation method (eCIM) according to Clinical and Laboratory Standards Institute (CLSI) guidelines. mCIM was used to confirm phenotypic carbapenemase production, and eCIM was used to identify metallo-β-lactamase activity among mCIM-positive isolates. The assays were performed using meropenem disks and *Escherichia coli* ATCC 25922 as the indicator strain, with interpretation based on CLSI zone-diameter criteria. Detailed phenotypic confirmation procedures are provided in Supplemental method S1.1.

WGS was performed on all isolates in the discovery cohort for species identification, multilocus sequence typing, and antimicrobial resistance gene annotation. Genomic DNA was extracted from overnight cultures, and paired-end Illumina sequencing was performed using a PE150 strategy. Reads were assembled using SOAPdenovo v2.04 and subjected to gap closure with GapCloser v1.12; species identification, MLST assignment, and acquired resistance gene annotation were subsequently performed using Kleborate v3.2.4. Acquired resistance genes were considered confidently detected when the alignment met the default Kleborate thresholds of ≥90% nucleotide identity and ≥80% nucleotide coverage (15). For core-genome analysis, core genes were identified using Roary v3.12 with a BLASTP identity cutoff of 90%, recombinant regions were removed using Gubbins v2.2.0, and a maximum-likelihood phylogenetic tree was inferred using FastTree v2.1.7 (16, 17). Core-genome phylogeny was used to describe genetic relatedness and sequence type diversity. No pairwise SNP-distance cutoff was used to define genomic clusters or infer transmission, as the analysis was not intended for transmission clustering. Detailed WGS bioinformatics procedures are provided in Supplemental method S1.2.

In the workflow-evaluation cohort, common carbapenemase gene groups were detected using an in-house SYBR Green-based real-time PCR assay targeting *bla*_KPC_, *bla*_NDM_, *bla*_IMP_, *bla*_VIM_, and *bla*_OXA-48-like_ in separate singleplex reactions. DNA was prepared by boiling extraction, and a positive result required a cycle threshold (Ct) value <35 together with a single melting peak consistent with the corresponding genotype-confirmed positive control. Additional information on primer sources, reaction conditions, controls, and repeat-testing criteria is provided in Supplemental method S1.3.

### Comparative analysis of carbapenem susceptibility results

VITEK 2 AST was performed using AST-N335 (REF 418985) in the discovery cohort and AST-N334 (REF 418984) in the workflow-evaluation cohort. Both card configurations contain the same imipenem formulation (ipm04n). To assess whether the observed IPM-predominant discrepancy was confined to these card configurations or to this imipenem formulation, 29 clinical *Klebsiella pneumoniae* complex isolates and one quality-control strain (Escherichia coli ATCC 25922) were additionally retested using AST-N399 (REF 423701), which contains the reformulated imipenem formulation (ipm05n). The clinical subset included 19 previously confirmed *bla*_IMP-4_-positive IPM-discrepant isolates and 10 comparator isolates, comprising five *bla*_IMP-4_-positive non-discrepant isolates, one *bla*_IMP-26_-positive isolate, one *bla*_IMP-4_ +*bla*_KPC-2_ co-producing isolate, two *bla*_KPC-2_-positive isolates, and one *bla*_NDM-1_-positive isolate (Table S1).

BMD was used as the reference method for IPM and MEM MIC determination. BMD was performed according to CLSI broth microdilution principles using cation-adjusted Mueller-Hinton broth. Briefly, bacterial suspensions were prepared from overnight cultures and adjusted to a final inoculum of approximately 5 × 10^5^ CFU/mL in each well. Two-fold dilutions of IPM and MEM were tested over a concentration range of 0.016–32 mg/L. Plates were incubated at 35°C in ambient air for 16–20 h, and the MIC was read as the lowest antimicrobial concentration that completely inhibited visible growth. Essential agreement was defined as MIC results within ±1 log_2_ dilution of the BMD value. An AST discrepancy was defined as an MIC difference of ≥2 log_2_ dilutions between VITEK 2 and BMD.

Discrepancy adjudication differed by cohort. In the discovery cohort, isolates showing a ≥2 log_2_ MIC difference and/or major categorical disagreement underwent triplicate repeat testing, and a discrepancy was considered confirmed only when it persisted in at least two of three repeat tests. In the workflow-evaluation cohort, isolates lacking essential agreement were retested once, and the discrepancy was retained only if the ≥2 log_2_ dilution difference persisted in the repeat test.

### Exploratory phenotypic assays

To explore phenotypic features associated with the observed discordance, a low-inoculum spread-plate colony recovery assay was performed in a representative subpanel of 27 isolates selected from the discovery and workflow-evaluation cohorts. This subpanel included isolates producing IMP-4, NDM, OXA-48-like, KPC, and VIM carbapenemases, together with a carbapenemase-negative control isolate.

Briefly, bacterial suspensions prepared from overnight cultures were adjusted to 0.5 McFarland and serially diluted 10,000-fold. This inoculum was selected based on preliminary optimization experiments that yielded 50–300 CFU on antibiotic-free control plates. A 50-μL aliquot of the diluted suspension was spread onto paired antibiotic-free Mueller-Hinton agar and Mueller-Hinton agar containing imipenem or meropenem at 1, 2, or 4 mg/L, followed by incubation at 35°C for 18–24 h. Colonies were enumerated after incubation. Recovery across the concentration series was used to characterize differences in growth recovery between imipenem- and meropenem-containing media.

The Hydrolysis Discordance Index (HDI) was defined as the difference in relative recovery between MEM and IPM at 1 mg/L and was calculated as HDI = R_MEM,1 − R_IPM,1, where R_MEM,1 = N_MEM,1/N_control and R_IPM,1 = N_IPM,1/N_control. Here, N_MEM,1 and N_IPM,1 represent colony counts on meropenem- and imipenem-containing agar at 1 mg/L, respectively, and N_control represents the colony count on the paired antibiotic-free Mueller-Hinton control plate.

Following the initial concentration-series screening experiment, repeatability of the 1-mg/L colony-recovery assay was assessed in three independent repeat experiments performed on separate days, with duplicate plates for each isolate under each condition in each experiment. Intra-assay variability was assessed using the relative percent difference between duplicate plates, whereas between-experiment variability was summarized using the mean, standard deviation, and range of HDI values, and rank consistency across experiments was evaluated by pairwise Spearman correlation. Detailed isolate-level colony counts, relative-recovery values, HDI calculations, and variability metrics are provided in Supplemental method S1.4 and Table S2. The assay was considered an exploratory colony-recovery readout rather than a direct measure of carbapenem hydrolysis or enzyme kinetics.

### Phenotype-guided confirmatory workflow

Based on the phenotypic profile observed in the discovery cohort, we developed a targeted confirmatory workflow incorporating low-level carbapenem resistance and retained susceptibility to selected non-carbapenem agents. The screening criteria flagged isolates for further review when either of the following features was present: a reported IPM MIC of 2–8 mg/L, or an atypical carbapenem profile characterized by elevated IPM MICs with retained susceptibility to aztreonam, amikacin, and/or trimethoprim-sulfamethoxazole. Isolates meeting these criteria underwent genotypic confirmation by in-house PCR or lateral flow assay, followed by BMD when an IMP-type genotype was identified.

Instrument-flagged unreliable results were analyzed separately from unflagged discrepancies because flagged results would ordinarily prompt further review in routine laboratory practice. The primary practical outcome was recognition of unflagged ≥2 log_2_ MIC discrepancies between VITEK 2 and BMD. Workflow performance was calculated in a refined combined data set after excluding instrument-flagged unreliable results. True positives were isolates meeting workflow criteria and showing confirmed unflagged ≥2 log_2_ MIC discrepancies; false positives were isolates meeting workflow criteria without a confirmed unflagged discrepancy; false negatives were isolates not meeting workflow criteria but showing a confirmed unflagged discrepancy; and true negatives were isolates not meeting workflow criteria and without a confirmed unflagged discrepancy.

### Statistical analysis and figure preparation

Statistical analyses and data visualization were performed in Python version 3.9. Categorical variables, including susceptibility rates, were compared using the chi-squared test or Fisher’s exact test, as appropriate. For antimicrobial susceptibility profile comparisons, resistant isolates were compared with non-resistant isolates, where non-resistant was defined as susceptible plus intermediate. HDI values across genotype groups were summarized descriptively as part of an exploratory phenotypic analysis. Associations of confirmed carbapenem AST discrepancy with *bla*_IMP-4_ carriage and clinical department, and of IPM discrepancy with ESBL gene carriage and annotated OmpK35/OmpK36 alterations, were assessed in the discovery cohort using the chi-squared test or Fisher’s exact test, as appropriate. Conventional multivariable logistic regression including *bla*_IMP-4_ was not reliably estimable because all IPM-discrepant isolates carried *bla*_IMP-4_, resulting in quasi-complete separation. As an exploratory sensitivity analysis, multivariable logistic regression including ESBL gene carriage and annotated OmpK35/OmpK36 alterations was performed within isolates carrying *bla*_IMP-4_ as the sole detected carbapenemase gene. Workflow performance was summarized using sensitivity, specificity, positive predictive value, negative predictive value, and overall accuracy, with 95% confidence intervals calculated using the Wilson score method. A two-sided *P* value < 0.05 was considered statistically significant. Heatmaps, boxplots, and scatter plots were generated using Matplotlib and Seaborn. For Fig. 5, panel A was created using BioRender, whereas panels B–D were created from the underlying study data using Microsoft PowerPoint. Final figure assembly was performed in Microsoft PowerPoint.

## RESULTS

### Carbapenem susceptibility discordance was predominantly associated with imipenem

Among the 133 carbapenemase-producing *Klebsiella pneumoniae* complex isolates in the discovery cohort, 29 showed confirmed carbapenem AST discrepancies after repeat adjudication. VITEK 2 showed higher essential agreement with broth microdilution (BMD) for meropenem (MEM) than for imipenem (IPM) (95.49% versus 78.95%). Of the 29 discrepant isolates, 28 involved IPM and six involved MEM: 23 showed IPM-only discordance, five showed discordance for both agents, and one showed MEM-only discordance.

**Fig 1:**
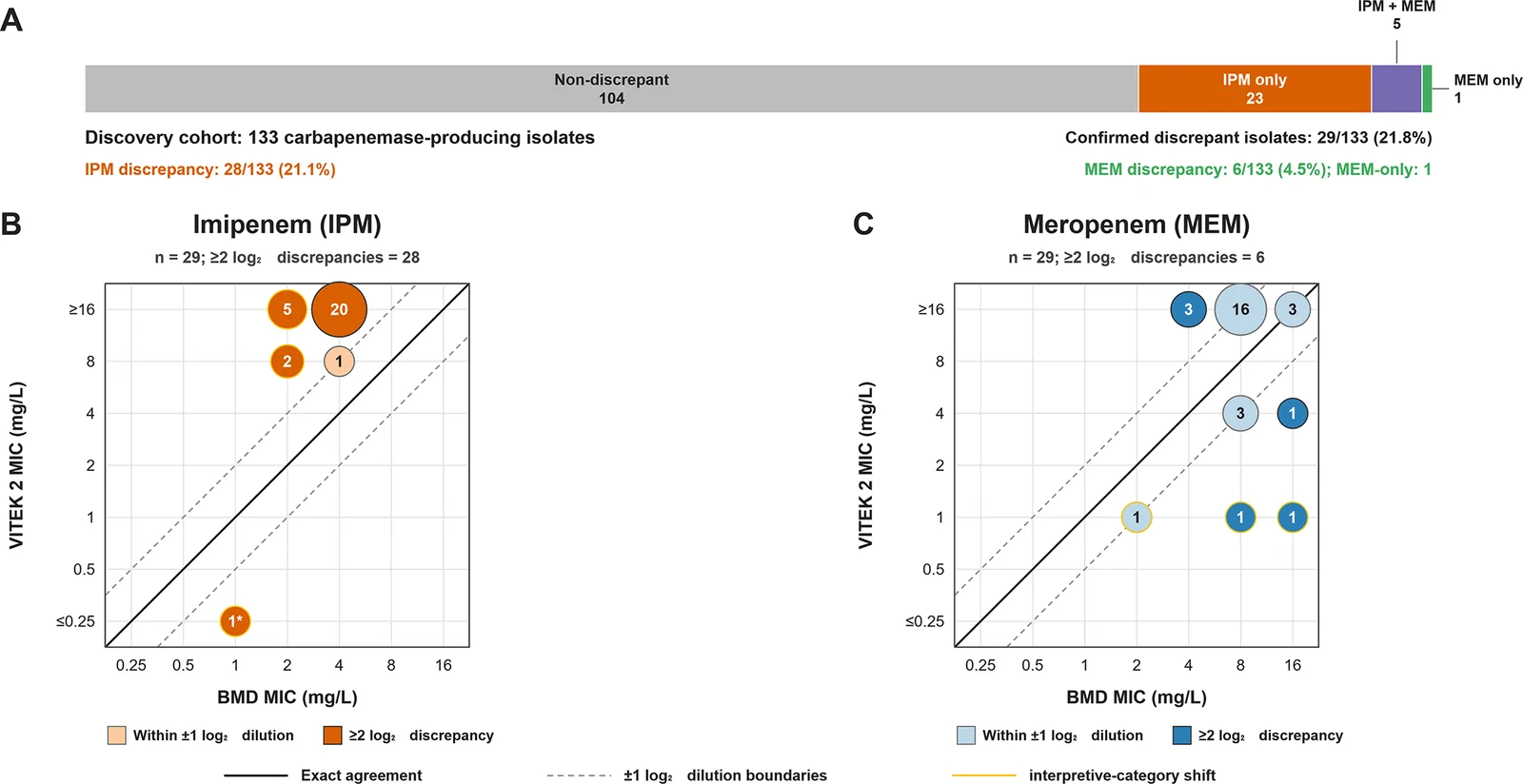
IPM-predominant carbapenem susceptibility discordance between VITEK 2 and BMD in the discovery cohort. (**A**) Distribution of 104 non-discrepant and 29 confirmed discrepant isolates, including 23 IPM-only, 5 concurrent IPM and MEM, and 1 MEM-only discrepancy. (**B and C**) Paired BMD and VITEK 2 MICs for IPM and MEM, respectively, among the 29 discrepant isolates. Bubble size and numbers indicate isolate counts; dark fill indicates a confirmed ≥ 2 log₂ MIC discrepancy, and gold outlines indicate interpretive-category shifts. Solid and dashed diagonal lines represent exact agreement and the ±1 log₂ dilution boundaries, respectively. Using CLSI breakpoints (susceptible, ≤1 mg/L; intermediate, 2 mg/L; resistant, ≥4 mg/L), IPM showed seven intermediate-to-resistant shifts and one AES-related susceptible-to-resistant shift (asterisk; VITEK 2 MIC ≤0.25 mg/L but final category resistant). MEM showed two resistant-to-susceptible shifts and one intermediate-to-susceptible shift; the latter did not meet the confirmed MEM MIC-discrepancy criterion.

For IPM, 27 of the 28 discordant isolates had raw VITEK 2 MICs at least 2 log_2_ dilutions higher than the corresponding BMD MICs. The remaining IPM-discrepant isolate had a low raw MIC but was reported as resistant after Advanced Expert System (AES) adjustment. Using CLSI breakpoints, interpretive-category shifts occurred in 8 of the 28 IPM-discrepant isolates (28.6%). Of these, seven were intermediate by BMD but resistant by VITEK 2, whereas the remaining isolate was susceptible by BMD but resistant according to the final VITEK 2 report. For MEM, interpretive-category shifts occurred in 3 of the 29 discrepant isolates (10.3%) and were all toward a lower VITEK 2 category. Of these, two isolates were resistant by BMD but susceptible by VITEK 2, and one was intermediate by BMD but susceptible by VITEK 2. Two of these three isolates also met the confirmed MEM MIC-discrepancy criterion. The drug-level discrepancy profile is shown in Fig. 1A, and the corresponding MIC distributions relative to CLSI breakpoints are shown in Fig. 1B and C. Categorical error rates are summarized in Table 1.

**TABLE 1:** Agreement and discrepancy profile of VITEK 2 compared with BMD for carbapenem MIC determination in the discovery cohort^*b*^^,^^*c*^

Antibiotic	CA, *n*/*N* (%)	EA, *n*/*N* (%)	Confirmed AST discrepancy, *n*/*N* (%)	Direction or source of confirmed discrepancy	mE, *n*/*N* total (%)	ME, *n*/*N* BMD-S (%)	VME, *n*/*N* BMD-R (%)
MEM	130/133 (97.74)	127/133 (95.49)	6/133 (4.51)	Higher raw VITEK 2 MIC, 3/6; lower raw VITEK 2 MIC, 3/6	1/133 (0.75)	NE	2/132 (1.52)
IPM	125/133 (93.98)	105/133 (78.95)	28/133 (21.05)	Higher raw VITEK 2 MIC, 27/28; AES-adjusted categorical discrepancy, 1/28	7/133 (5.26)	1/2 (50.0)^*a*^	0/124 (0.00)

^*a*^ Based on two BMD-susceptible isolates.

^*b*^ NE, not estimable because no isolate was susceptible by BMD.

^*c*^ AES, Advanced Expert System; BMD, broth microdilution; CA, categorical agreement; EA, essential agreement; IPM, imipenem; MEM, meropenem; mE, minor error; ME, major error; VME, very major error. EA was defined as agreement within ±1 log₂ dilution between raw VITEK 2 and BMD MICs. CA and categorical errors were based on CLSI breakpoints and final VITEK 2 interpretations, including AES adjustments. Confirmed AST discrepancy comprised a repeat-confirmed raw MIC difference of ≥2 log₂ dilutions or one AES-related IPM major error. mE, ME, and VME rates used all isolates, BMD-susceptible isolates, and BMD-resistant isolates as denominators, respectively.

To assess the cross-card consistency of the observed IPM elevation, 29 selected isolates were retested with AST-N399, including 19 previously IPM-discrepant IMP-4-producing isolates and 10 comparator isolates. All 19 previously discrepant isolates again yielded IPM MICs above the corresponding BMD values. Of these 19 isolates, 11 retained a ≥2 log2 discrepancy, whereas eight showed a one-dilution elevation. Among the 10 comparator isolates, 2 IMP-4-producing isolates newly met the ≥2 log_2_ discrepancy criterion. These findings indicate that the direction of IPM elevation was reproduced with AST-N399 in the selected previously discrepant isolates, although the magnitude of the difference varied (Table S1).

### Discrepant isolates showed relatively low carbapenem MICs by BMD and lower resistance to selected non-carbapenem agents

**Fig 2:**
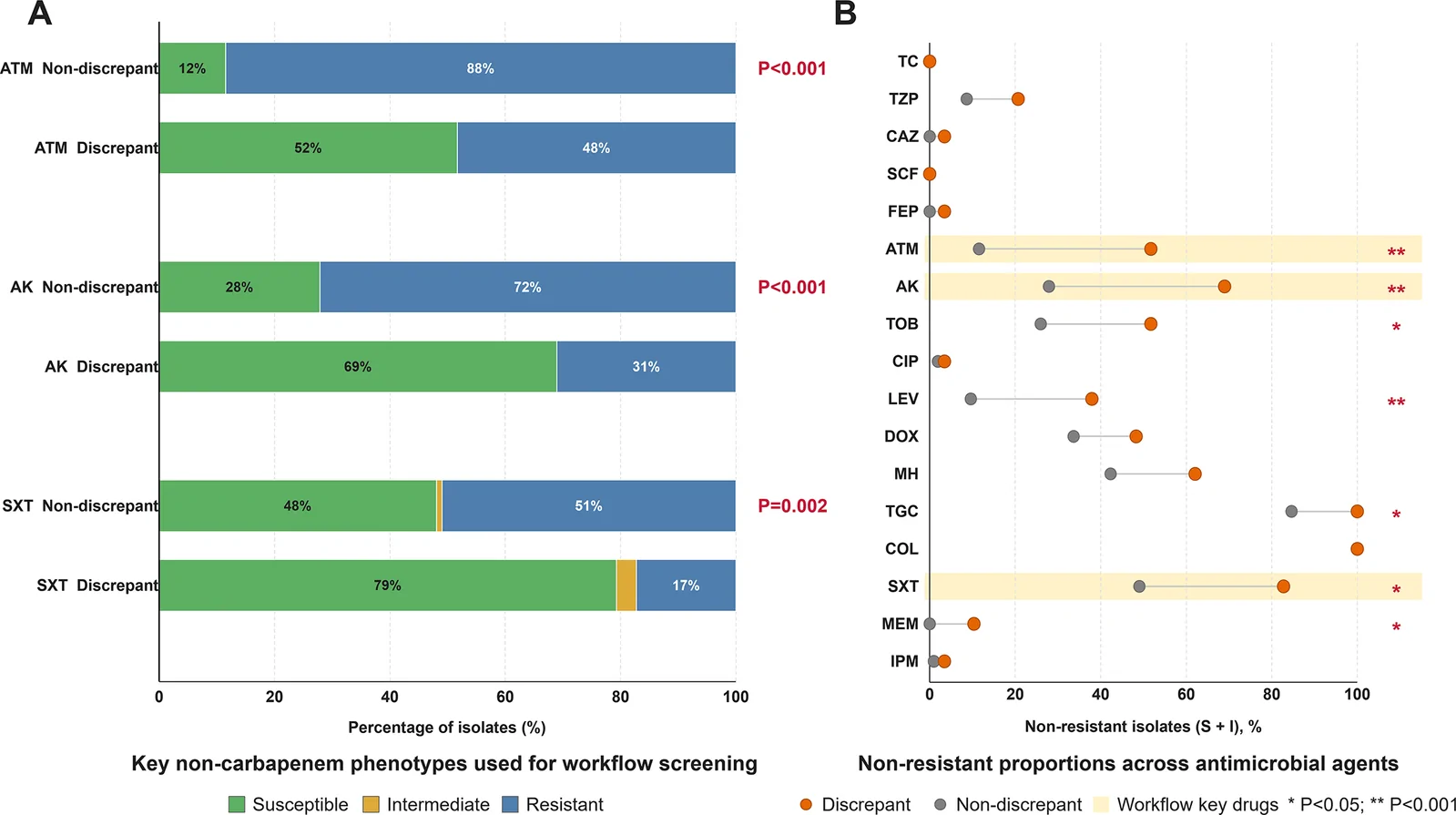
Phenotypic signature of AST-discrepant isolates. (**A**) Susceptible, intermediate, and resistant proportions for aztreonam, amikacin, and trimethoprim-sulfamethoxazole in discrepant and non-discrepant isolates. These three non-carbapenem agents were selected because retained susceptibility to these agents contributed to the phenotype-guided screening strategy. (**B**) Non-resistant proportions, defined as susceptible plus intermediate (S + I), across 17 antimicrobial agents in discrepant and non-discrepant isolates. Highlighted rows indicate antimicrobial agents incorporated into the workflow criteria. Statistical comparisons were made between resistant and non-resistant proportions. Chi-squared or Fisher’s exact tests were used as appropriate according to expected cell counts. Asterisks indicate statistical significance.

The BMD MICs of the 29 discrepant isolates were most frequently 8 mg/L for MEM and 4 mg/L for IPM (Fig. 1B and C). Compared with non-discrepant isolates, discrepant isolates more frequently retained susceptibility to selected non-carbapenem agents. For between-group statistical comparisons, susceptible and intermediate interpretations were combined as non-resistant and compared with resistant interpretations. Compared with non-discrepant isolates, discrepant isolates were more frequently non-resistant to aztreonam (15/29 [51.7%] versus 12/104 [11.5%]; *P* < 0.001), amikacin (20/29 [69.0%] versus 29/104 [27.9%]; *P* < 0.001), and trimethoprim-sulfamethoxazole (23/29 [79.3%] versus 50/104 [48.1%]; *P* = 0.002) (Fig. 2). Together, these findings defined a recognizable lower-resistance profile that was subsequently incorporated into the phenotype-guided screening workflow. The complete 17-agent susceptibility profile and the corresponding statistical comparisons are provided in Fig. S1.

### The discrepant phenotype was strongly associated with *bla*_IMP-4_ and was distributed across multiple lineages

WGS showed that all 29 discrepant isolates carried *bla*_IMP-4_ as the sole detected carbapenemase gene. In contrast, *bla*_IMP-4_ alone was detected in only 14 of 104 non-discrepant isolates, and one additional non-discrepant isolate co-carried *bla*_KPC-2_ and *bla*_IMP-4_. Overall, confirmed carbapenem AST discrepancy was observed in 29 of 44 *bla*_IMP-4_-positive isolates (65.9%), compared with none of the 89 *bla*_IMP-4_-negative isolates (*P* < 0.001). *bla*_KPC-2_ predominated among non-discrepant isolates, accounting for 77 of 104 isolates. No confirmed discrepancy was observed among isolates carrying *bla*_KPC-2_ alone, *bla*_NDM-1_, or *bla*_IMP-26_, or in the single isolate co-carrying *bla*_KPC-2_ and *bla*_IMP-4_ (Table 2). Genotype-stratified BMD MIC distributions for IPM and MEM are shown in Fig. S2.

**TABLE 2:** Carbapenemase genotype distribution and AST discrepancy status in the discovery cohort^*c*^

Carbapenemase genotype	Total isolates, *n*/*N* (%)	Discrepant isolates, *n*/*N* (%)	Non-discrepant isolates, *n*/*N* (%)	Discrepancy rate within genotype, *n*/*N* (%)	IPM discrepancy, *n*	MEM discrepancy, *n*
KPC-2	77/133 (57.9)	0/29 (0.0)	77/104 (74.0)	0/77 (0.0)	0	0
IMP-4	43/133 (32.3)	29/29 (100.0)	14/104 (13.5)	29/43 (67.4)	28^*b*^	6^*b*^
NDM-1	9/133 (6.8)	0/29 (0.0)	9/104 (8.7)	0/9 (0.0)	0	0
IMP-26	3/133 (2.3)	0/29 (0.0)	3/104 (2.9)	0/3 (0.0)	0	0
KPC-2 + IMP-4^*a*^	1/133 (0.8)	0/29 (0.0)	1/104 (1.0)	0/1 (0.0)	0	0
Total	133/133 (100.0)	29/29 (100.0)	104/104 (100.0)	29/133 (21.8)	28	6

^*a*^ The KPC-2+IMP-4 row denotes one isolate co-carrying both *bla*_KPC-2_ and *bla*_IMP-4_; this isolate was non-discrepant in the discovery cohort.

^*b*^ IPM and MEM discrepancy counts were not mutually exclusive because five isolates showed discrepancies for both agents.

^*c*^ AST, antimicrobial susceptibility testing; IPM, imipenem; MEM, meropenem.

**Fig 3:**
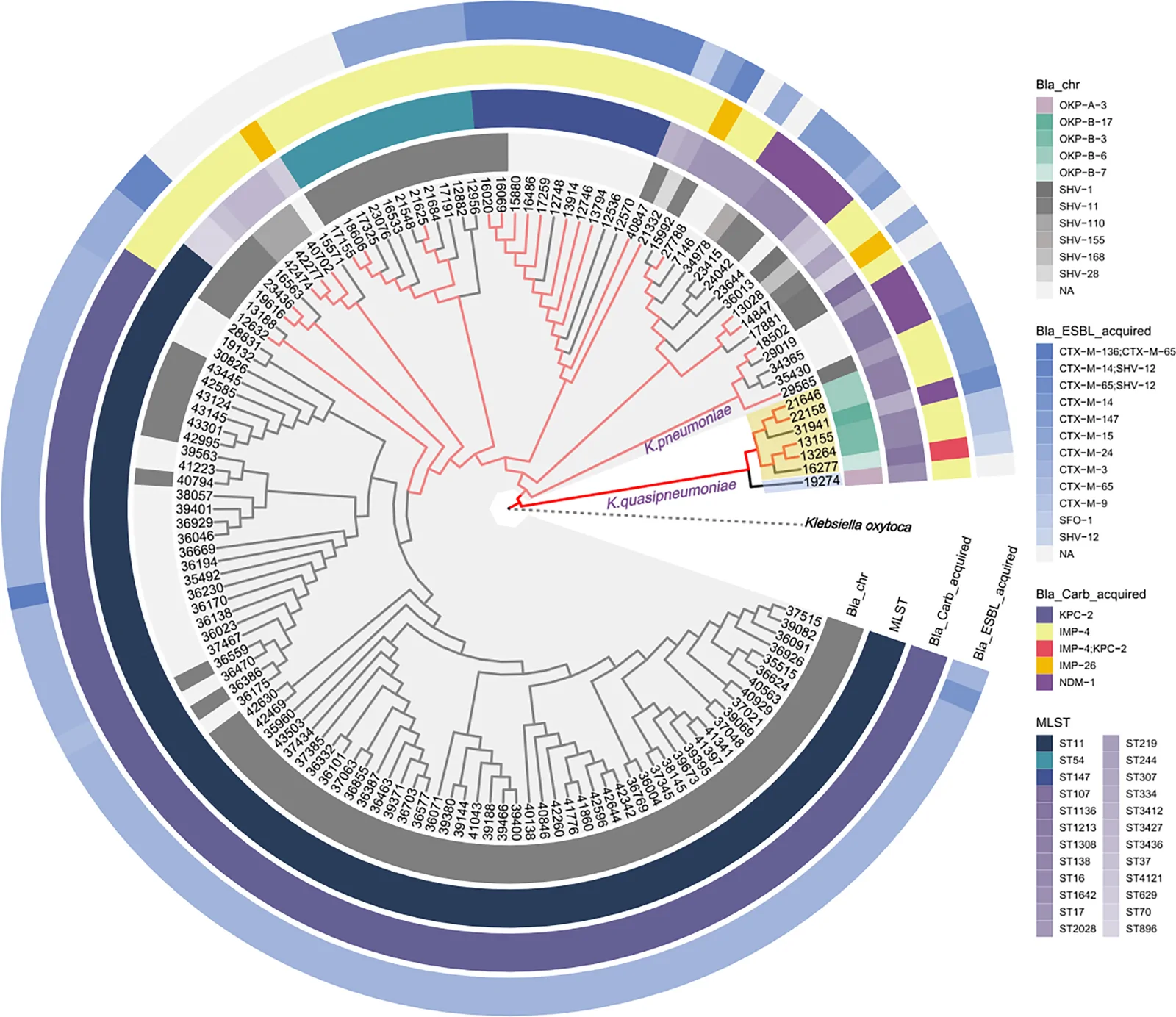
Phylogenetic distribution of AST-discrepant isolates across the *Klebsiella pneumoniae* complex. Maximum-likelihood phylogeny based on core-genome single-nucleotide polymorphisms among the 133 carbapenemase-producing isolates in the discovery cohort. Branches or labels corresponding to AST-discrepant isolates are highlighted in red, whereas non-discrepant isolates are shown in gray. Background shading indicates species or subspecies assignment within the *Klebsiella pneumoniae* complex, including *K. pneumoniae, K. quasipneumoniae* subsp. *similipneumoniae*, and *K. quasipneumoniae* subsp. *quasipneumoniae*. The outer annotations show sequence type and selected resistance gene profiles. The distribution of discrepant isolates across multiple sequence types indicates that the discrepant phenotype was not restricted to a single clonal lineage.

Core-genome phylogeny showed that discrepant isolates were distributed across multiple phylogenetic branches (Fig. 3). Species-level assignment by WGS identified 25 of the 29 discrepant isolates as *K. pneumoniae* and four as *K. quasipneumoniae* subsp. *similipneumoniae*. Multilocus sequence typing (MLST) revealed 14 sequence types, with ST147 being the most frequent, followed by ST54 and ST3427. Discrepant isolates were more frequently recovered from the Neonatology department than non-discrepant isolates (13/29 [44.8%] versus 8/104 [7.7%]; *P* < 0.001). Nevertheless, their distribution across two taxa, 14 sequence types, and multiple phylogenetic branches indicated that the phenotype was not restricted to a single lineage. Clinical metadata, specimen-source distribution, and isolate-level resistance gene profiles are provided in Table S3.

Across the overall discovery cohort, extended-spectrum β-lactamase (ESBL) gene carriage was more frequent among IPM-discrepant isolates than among isolates without IPM discrepancy (20/28 [71.4%] versus 31/105 [29.5%]; *P* < 0.001), whereas annotated OmpK35/OmpK36 sequence alterations were less frequent (11/28 [39.3%] versus 81/105 [77.1%]; *P* < 0.001). However, because the overall cohort included multiple carbapenemase backgrounds, these comparisons could be confounded by carbapenemase type. Therefore, further analysis was restricted to the 43 isolates carrying *bla*_IMP-4_ as the sole detected carbapenemase gene. Within this subgroup, ESBL gene carriage was detected in 20 of 28 (71.4%) IPM-discrepant isolates and 4 of 15 (26.7%) isolates without IPM discrepancy, whereas annotated OmpK35/OmpK36 sequence alterations were detected in 11 of 28 (39.3%) and 2 of 15 (13.3%) isolates, respectively. In an exploratory multivariable logistic regression model including both annotation-derived variables, ESBL gene carriage showed an adjusted association with IPM discrepancy (adjusted OR, 6.19; 95% CI, 1.04–36.78; *P* = 0.045), whereas annotated OmpK35/OmpK36 sequence alterations were not associated with IPM discrepancy (adjusted OR, 1.22; 95% CI, 0.14–10.48; *P* = 0.855).

### Exploratory colony-recovery assays showed heterogeneous IPM–MEM recovery patterns

To explore phenotypic behavior potentially related to the relatively weak and substrate-dependent carbapenemase activity proposed for IMP-4, we assessed low-inoculum recovery under IPM and MEM exposure in a selected panel of 27 isolates from the discovery and workflow-evaluation cohorts. The panel included 10 IMP-4, 5 NDM, 4 OXA-48-like, 4 KPC, and 2 VIM producers, together with 1 KPC-33 comparator and one carbapenemase-negative control. The HDI was used as an exploratory phenotypic index of MEM-over-IPM relative recovery at 1 mg/L and not as a direct measurement of carbapenem hydrolysis.

**Fig 4:**
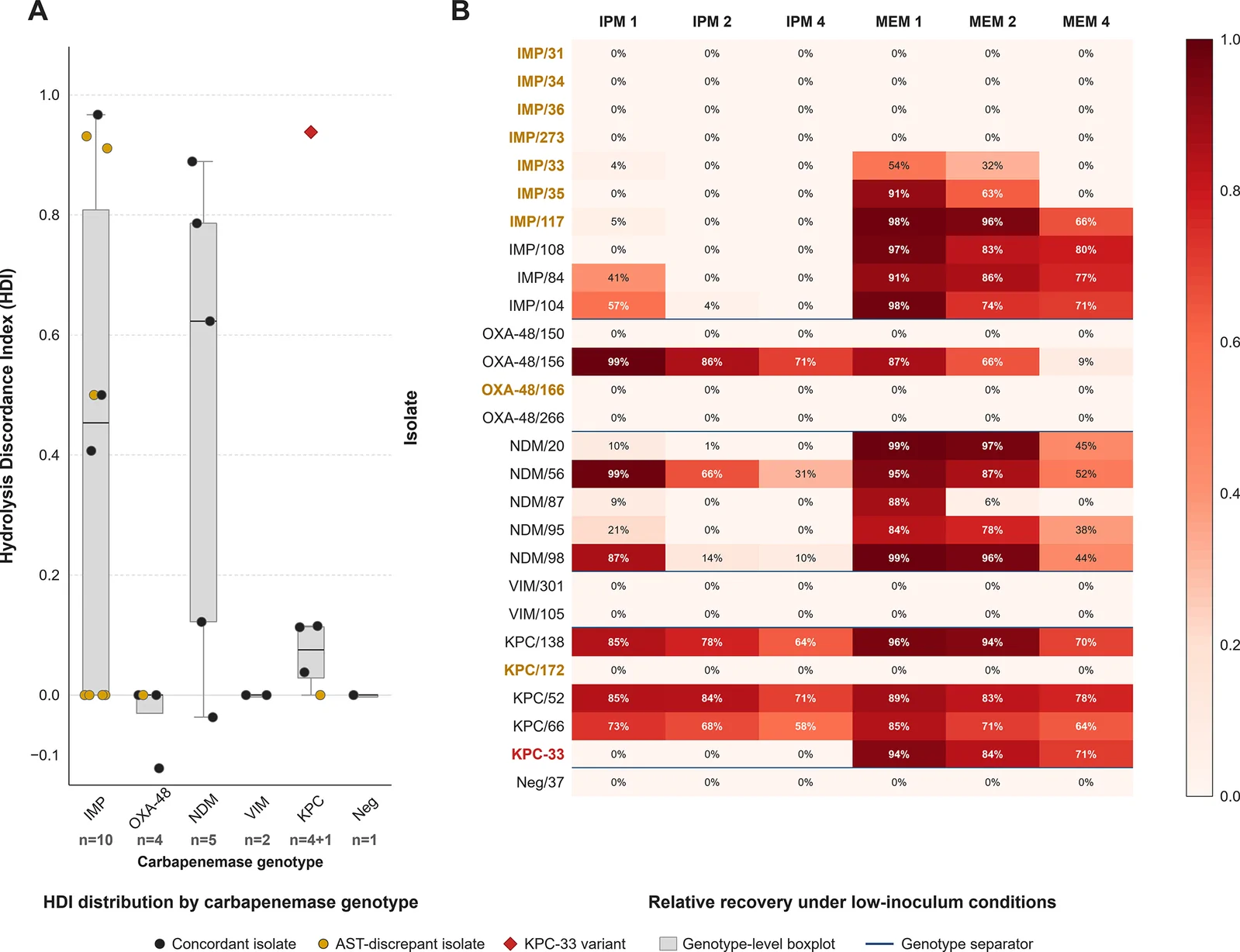
Exploratory phenotypic assessment of substrate-specific and density-dependent recovery. (**A**) Distribution of the Hydrolysis Discordance Index (HDI) across carbapenemase genotypes. Individual isolates are overlaid on genotype-level boxplots. AST-discrepant isolates are highlighted in gold, concordant isolates in black, and the KPC-33 variant in red. KPC-33 was included as a comparator with carbapenem-specific phenotypic discordance and was excluded from the wild-type KPC baseline boxplot. HDI was calculated as the difference in relative recovery between MEM and IPM at 1 mg/L. (**B**) Heatmap showing relative recovery rates under low-inoculum conditions at IPM and MEM concentrations of 1, 2, and 4 mg/L. Values were normalized to antibiotic-free growth controls. Darker red indicates higher relative recovery. Strain labels highlighted in gold indicate isolates with confirmed AST discrepancies, and the KPC-33 variant is highlighted in red. Horizontal separator lines indicate carbapenemase genotype groups.

In the initial concentration-series experiment, the 10 IMP-4 isolates showed a broad distribution of HDI values, ranging from near 0 to 0.97 (Fig. 4A). Among the 10 IMP-4 isolates, 4 showed no recovery under any of the tested carbapenem conditions, whereas all 6 isolates with detectable recovery showed greater recovery on MEM- than on IPM-containing agar (Fig. 4B). Elevated MEM-over-IPM recovery was also observed in several NDM isolates and the KPC-33 comparator, whereas most KPC and OXA-48-like isolates showed low HDI values. Thus, elevated HDI was not specific to IMP-4.

Following the initial screening experiment, repeatability was evaluated in three independent repeat experiments performed on separate days, with duplicate plates for each condition in each experiment. Median duplicate-plate relative percent differences were 13.6%, 0%, and 6.0% for the antibiotic-free control, IPM, and MEM conditions, respectively. HDI values across the three repeat experiments showed moderate-to-strong pairwise rank correlations (Spearman ρ = 0.627–0.786; all *P* < 0.001). However, the initially elevated HDI values were not reproduced across the three repeat experiments for 2 of the 27 isolates (7.4%), comprising one IMP-4-producing isolate and one NDM-producing isolate. Detailed colony counts, relative recovery values, HDI calculations, and repeatability metrics are provided in Table S2.

### A phenotype-guided workflow identified unflagged MIC discrepancies

**Fig 5:**
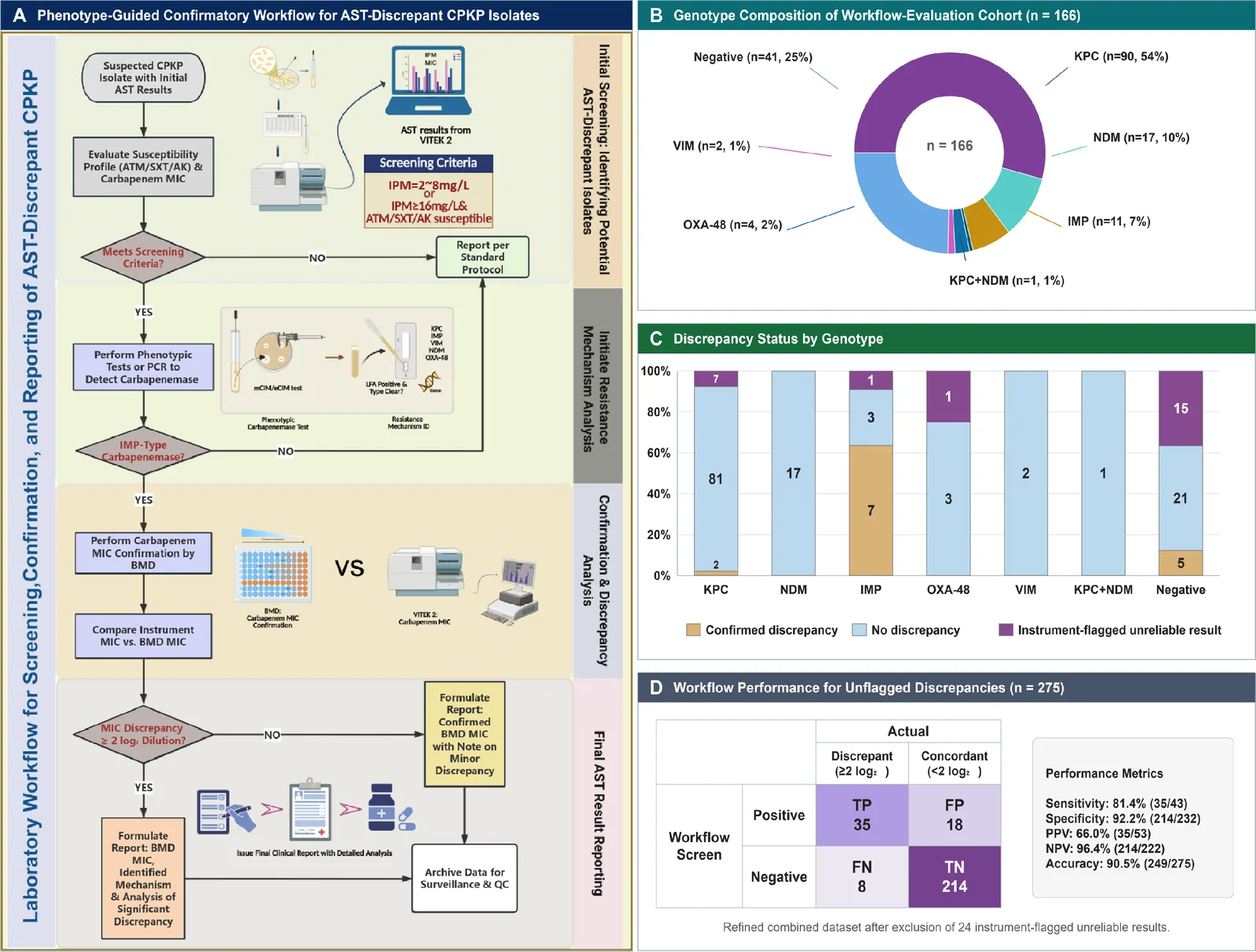
Phenotype-guided confirmatory workflow and performance evaluation. (**A**) Proposed laboratory workflow for screening, confirming, and reporting AST discrepancies in suspected carbapenemase-producing *Klebsiella pneumoniae* complex isolates. (**B**) Genotype composition of the 166-isolate workflow-evaluation cohort. (**C**) Distribution of discrepancy status by genotype in the workflow-evaluation cohort. Results are categorized as confirmed unflagged discrepancy, no discrepancy, or instrument-flagged unreliable result. (**D**) Confusion matrix and performance metrics of the phenotype-guided workflow in the refined combined data set (*n* = 275), using unflagged AST discrepancy as the target outcome. Panel **A** was created in BioRender. Lin, R. (2026) https://BioRender.com/ryfzeou.

Based on the relatively low carbapenem MICs, lower resistance to selected non-carbapenem agents, and the strong association with *bla*_IMP-4_, we developed a phenotype-guided confirmatory workflow for targeted follow-up testing (Fig. 5A). The workflow was evaluated in a retrospectively enriched cohort of 166 *K. pneumoniae* isolates collected between 2018 and 2024. In this cohort, VITEK 2 showed an IPM essential agreement of 77.1% with BMD, and 38 isolates had IPM results requiring adjudication, including 24 instrument-flagged unreliable results and 14 unflagged ≥2 log_2_ MIC discrepancies from BMD.

Genotype distributions differed according to discrepancy type. Among the 24 instrument-flagged isolates, 15 were carbapenemase-negative, 7 carried KPC-type carbapenemase, 1 carried OXA-48-like, and 1 carried an IMP-type carbapenemase. Among the 14 isolates with unflagged ≥2 log₂ discrepancies, 7 carried IMP-type carbapenemases, 5 were carbapenemase-negative, and 2 carried KPC-type carbapenemases. Because instrument-flagged results would ordinarily prompt additional review, unflagged discrepancies were treated as the primary practical target of the workflow (Fig. 5B and C).

In a refined combined data set of 275 isolates after excluding instrument-flagged unreliable results, the phenotype-guided workflow identified unflagged ≥2 log_2_ MIC discrepancies with a sensitivity of 81.4% (95% CI, 67.4%–90.3%), specificity of 92.2% (95% CI, 88.1%–95.0%), positive predictive value of 66.0% (95% CI, 52.6%–77.3%), negative predictive value of 96.4% (95% CI, 93.1%–98.2%), and overall accuracy of 90.5% (95% CI, 86.5%–93.5%) (Fig. 5D). These estimates represent internal performance within the retrospectively enriched study data set and should not be generalized to routine laboratory populations. The genotype-status counts and performance calculations are provided in Table S4.

## DISCUSSION

Carbapenem-resistant *Klebsiella pneumoniae* complex isolates remain a major challenge for antimicrobial therapy and infection control, and accurate laboratory recognition of resistance phenotypes is essential for both clinical management and surveillance (1–4). Previous studies have documented low-level carbapenem phenotypes and method-dependent AST variability among carbapenemase-producing Enterobacterales, including OXA-48-like and metallo-β-lactamase-producing isolates (5, 7, 12, 13, 18–20). Against this background, we identified a repeat-confirmed, *bla*_IMP-4_-associated discordance pattern between VITEK 2 and BMD that predominantly involved IPM. In most IPM-discrepant isolates, VITEK 2 yielded higher raw IPM MICs than BMD, while one isolate showed a distinct AES-adjusted resistant interpretation despite a low raw MIC. Although only a subset of the discrepant IPM results led to CLSI interpretive-category shifts, all such shifts were toward a higher VITEK 2 category, while many isolates remained resistant by both methods. The discrepant isolates also showed a recognizable low-level carbapenem profile by BMD and lower resistance to selected non-carbapenem agents, which provided the phenotypic basis for the proposed confirmatory workflow. Thus, the principal contribution of this study lies not in identifying a previously unrecognized low-level resistance phenomenon, but in characterizing a specific association among *bla*_IMP-4_, the observed phenotype, and the VITEK 2 platform that may guide targeted confirmatory testing and more cautious interpretation of automated carbapenem MIC results.

The laboratory significance of this discordance extends beyond categorical errors to the distortion of a recognizable low-level IMP-4-associated phenotype. Because VITEK 2 tended to report higher IPM MICs than BMD, the principal concern was not false susceptibility but upward shifts in IPM MICs and, in a subset of isolates, interpretive categories. Such overestimation may mask the relatively low-level carbapenem phenotype observed by BMD and reduce its phenotypic distinction from isolates with higher-level carbapenem resistance. This issue is also relevant to surveillance because IMP-4-producing *K. pneumoniae* has been reported to disseminate through clonal expansion and plasmid-mediated spread, and IMP carbapenemases are increasingly described across diverse geographic and genomic backgrounds (21–23). In the present study, discrepant isolates were distributed across multiple sequence types, two taxa within the *K. pneumoniae* complex, and several phylogenetic branches, indicating that the phenotype was not restricted to a single lineage. However, the phylogenetic analysis was not designed to infer or exclude local transmission. Discrepant isolates were also more frequently recovered from the Neonatology department (24–26), although this department-level enrichment was observational and requires validation in other populations and settings. Together, these findings support closer attention to platform-dependent MIC patterns during local surveillance and targeted confirmatory testing.

The exploratory low-inoculum colony-recovery assays provide hypothesis-generating context for the observed IPM-predominant discordance. IMP-type carbapenemases are biochemically diverse, and IMP-4 has been reported to exhibit substrate-dependent hydrolytic activity (27, 28). Earlier biochemical work on IMP-4, although performed mainly in *Acinetobacter* spp., suggested differential affinity toward meropenem and imipenem, supporting the possibility of substrate-associated carbapenem phenotypes (27). In the present study, HDI varied widely among IMP-4 isolates, and elevated MEM-over-IPM recovery was neither uniform within this group nor confined to IMP-4, as similar patterns were observed in several NDM-producing isolates and the KPC-33 comparator. KPC-33 has likewise been reported to display a higher MEM MIC than IPM MIC (29). Moreover, the initially elevated HDI values of two isolates were not reproduced across the three independent repeat experiments, indicating isolate-level experimental variability. Together, these findings are consistent with, but do not establish, substrate-associated phenotypic variability among carbapenemase-producing isolates. They therefore provide experimental context for the IPM-predominant discordance rather than a demonstrated mechanistic explanation. Accordingly, HDI should be interpreted as an exploratory relative-recovery index rather than a direct measure of carbapenem hydrolysis, and its biological significance requires validation using independent biochemical assays.

Beyond enzyme–substrate interactions, platform-specific testing conditions and isolate-level resistance backgrounds may also shape the observed discordance. Cross-card retesting with AST-N399 reproduced the direction of IPM elevation in the selected previously discrepant isolates, although the magnitude of the MIC difference varied and two *bla*_IMP-4_-positive comparator isolates newly crossed the ≥2 log₂ discrepancy threshold. Notably, a 2026 U.S. Food and Drug Administration (FDA) Class II recall (recall no. Z-1236-2026) included AST-N399 and other VITEK 2 Gram-negative susceptibility cards containing the ipm05n imipenem formulation because of the potential for quality-control failures and false-resistance results (https://www.accessdata.fda.gov/scripts/cdrh/cfdocs/cfRes/res.cfm?id=218083). This regulatory observation further highlights the potential contribution of formulation- or platform-related effects to imipenem susceptibility testing. Accordingly, our cross-card findings indicate that the observed IPM elevation was not confined to the original AST-N334/N335 card configurations, but they do not establish card independence. Inoculum effects and carbapenemase-associated changes in MIC determination have been described previously (12, 30, 31). Although the initial inoculum ranges used in VITEK 2 and BMD are broadly comparable, the enclosed microwell format and dynamic monitoring mode of automated systems differ from the larger, static broth environment of reference BMD. These differences may influence measured MICs by changing the experimental conditions under which bacterial growth and antimicrobial activity are assessed (30, 32). ESBL gene carriage and porin alterations were evaluated as components of the broader resistance background (32–34) rather than as established mechanistic determinants of the observed platform-specific discordance. Within the *bla*_IMP-4_-only subgroup, ESBL gene carriage showed an adjusted association with IPM discrepancy, whereas annotated OmpK35/OmpK36 sequence alterations did not. However, the small subgroup size, wide confidence interval, co-occurrence of the porin annotations with ESBL genes, and absence of functional validation limit mechanistic interpretation of these findings. In particular, the observed statistical association does not establish that ESBL gene carriage directly contributes to the discordance. Other unmeasured testing-related variables, including zinc concentrations in commercial media, may influence susceptibility testing of metallo-β-lactamase-producing Enterobacterales (35–37). Taken together, the observed discordance may reflect interactions among enzyme properties, isolate-level resistance backgrounds, and platform-specific testing conditions rather than the effect of a single determinant.

From a routine laboratory perspective, the phenotype-guided workflow is best interpreted as a locally adaptable reflex-testing filter for prioritizing confirmatory testing rather than a universal screening algorithm. Its practical target is the unflagged discrepancy. Instrument-flagged unreliable results would ordinarily trigger review, whereas unflagged ≥2 log₂ MIC differences may not prompt additional testing unless explicit review rules are in place. In the workflow-evaluation cohort, IMP-type isolates were the largest subgroup among unflagged discrepancies, while carbapenemase-negative isolates with borderline MICs predominated among instrument-flagged unreliable results, supporting separate interpretation of these categories. In the combined internal analysis set, the workflow achieved 81.4% sensitivity, 92.2% specificity, and a high negative predictive value of 96.4%. Within this retrospectively enriched study data set, these estimates suggest potential use for prioritizing confirmatory testing, while moderate positive predictive value indicates that isolates meeting the workflow criteria still require reference MIC testing and, where appropriate, molecular characterization. This approach complements but does not replace established carbapenemase screening. EUCAST emphasizes the importance of low-level carbapenem phenotypes, although implementation of low-threshold MIC criteria can be drug-, card-, and platform-dependent. Because the VITEK 2 MEM readout used in this study did not include a reportable 0.125-mg/L category, the EUCAST meropenem screening threshold could not be applied directly. Finally, because ATM/AK/SXT retained susceptibility is unlikely to be intrinsic to *bla*_IMP-4_ and more likely reflects local accessory resistomes, the phenotypic criteria require local calibration before use in other epidemiologic settings.

This study has several limitations. First, it was conducted in a single regional setting, and the observed neonatal enrichment may not reflect epidemiological patterns in other institutions. Second, the workflow-evaluation cohort was retrospectively enriched rather than prospectively assembled from consecutive isolates, and the performance analysis was based on a combined data set that also included the discovery cohort. The reported sensitivity, specificity, PPV, NPV, and accuracy therefore represent internal performance estimates and should not be generalized to routine laboratory populations. In particular, PPV and NPV are expected to vary with the prevalence of MIC discrepancies in the population tested. Third, the colony-recovery assays and WGS-derived resistance-background analyzes were exploratory, and HDI was not validated against an independent quantitative hydrolysis assay. These analyses therefore did not establish the biochemical or functional mechanism underlying the discordance. Finally, clinical treatment and outcome data were unavailable, so the observed MIC and interpretive-category shifts should be regarded as laboratory findings rather than evidence of clinical impact. Multicenter prospective validation using consecutive isolates and paired reference testing is required before broader implementation of the phenotype-guided workflow.

### Conclusion

In conclusion, this study characterizes a repeat-confirmed *bla*_IMP-4_-associated, IPM-predominant discordance pattern between VITEK 2 and BMD in *Klebsiella pneumoniae* complex isolates. The discrepant phenotype was characterized by relatively low carbapenem MICs by BMD, lower resistance to selected non-carbapenem agents, and heterogeneous IPM–MEM recovery patterns across multiple genetic lineages. These findings establish a laboratory-relevant genotype–phenotype–platform association and suggest that a phenotype-guided reflex-testing workflow may help identify unflagged MIC discrepancies and prioritize confirmatory testing. Prospective multicenter validation and local calibration are needed to define its applicability in routine laboratory practice

## Data Availability

Genome assemblies for all 133 isolates in the discovery cohort are publicly available through the NCBI under BioProject accession PRJNA793616. The isolate numbers used in the phylogenetic tree and Table S3.1 correspond directly to the NCBI strain identifiers after addition of the prefix “lkpn” (e.g., isolate 12536 corresponds to NCBI strain lkpn12536). BioProject PRJNA793616 also contains additional assemblies that were not included in the present discovery cohort.

## SUPPLEMENTAL MATERIAL

Supplemental figures spectrum.01493-26-s0001.docx Fig. S1 to S3.

Supplemental methods spectrum.01493-26-s0002.docx Supplemental methods.

Supplemental tables spectrum.01493-26-s0003.xlsx Tables S1 to S4.

ASM does not own the copyrights to Supplemental Material that may be linked to, or accessed through, an article. The authors have granted ASM a non-exclusive, world-wide license to publish the Supplemental Material files. Please contact the corresponding author directly for reuse.
