# Supplementary material for: blaIMP-4-associated imipenem susceptibility discordance in the Klebsiella pneumoniae complex: characterization and a phenotype-guided confirmatory workflow
Source: Microbiol Spectr. 2026 Oct 14;14(11):e01493-26. doi: 10.1128/spectrum.01493-26 (PMC13729153; doi:10.1128/spectrum.01493-26)
Supplement: Supplementary file 1 [file spectrum.01493-26-s0001.docx]

**Figure S1. Full antimicrobial susceptibility profiles of discrepant and non-discrepant isolates.
(A and B)** Stacked bar charts showing susceptible, intermediate, and resistant proportions for 17 antimicrobial agents among non-discrepant isolates (n = 104) and discrepant isolates (n = 29), respectively. Highlighted rows indicate the non-carbapenem agents incorporated into the phenotype-guided workflow. The right column shows statistical comparisons of resistant versus non-resistant proportions, where non-resistant was defined as susceptible plus intermediate. Fisher’s exact test or chi-square test was used according to expected cell counts. S, susceptible; I, intermediate; R, resistant.

**Figure S2. BMD carbapenem MIC distribution by carbapenemase genotype.**

Heatmap showing the within-genotype percentage distribution of meropenem (MEM) and imipenem (IPM) MICs determined by broth microdilution (BMD) in the discovery cohort. Each row represents one carbapenemase genotype–antibiotic combination, and each cell shows the percentage of isolates within that genotype falling into the corresponding MIC category. Darker blue indicates a higher within-genotype percentage. Percentages are rounded to whole numbers; row totals may therefore range from 98% to 101%

**Figure S3. Distribution of MEM and IPM MIC according to carbapenemase genotype and annotated *OmpK35*/36 status in the 2004–2017 CPKP cohort.** A) MEM and IPM MIC distributions for 133 CPKP isolates. Each circle represents the reported MEM or IPM MIC of one isolate and is colored according to the corresponding *OmpK35 and OmpK36* status identified by Kleborate from the genome assembly. Isolates are stratified by carbapenemase genotype, as indicated on x-axis. Wt indicates full-length *OmpK35* and *OmpK36* with no GD/TD insertion in the *OmpK36* β-strand loop; ▲ indicates missing/truncated porins;. GD indicates GD insertion in the *OmpK36* β-strand loop. The black dashed line indicates the CLSL non-susceptible breakpoint (MIC = 2 mg/L. B) IPM and MEM MIC values for AST discrepant and non-discrepant isolates stratified by carbapenemase genotype and annotated *OmpK35 and OmpK36* status. Porin-status definitions are as described in panel A.
