# Supplementary material for: blaIMP-4-associated imipenem susceptibility discordance in the Klebsiella pneumoniae complex: characterization and a phenotype-guided confirmatory workflow
Source: Microbiol Spectr. 2026 Oct 14;14(11):e01493-26. doi: 10.1128/spectrum.01493-26 (PMC13729153; doi:10.1128/spectrum.01493-26)
Supplement: Supplementary file 2 [file spectrum.01493-26-s0002.docx]

**Supplementary Methods**

**S1.1 Additional procedural details for mCIM and eCIM**

**S1.1.1 Modified carbapenem inactivation method**

For each isolate, a 1-μL loopful of growth from an overnight blood agar culture was emulsified in 2 mL of tryptic soy broth and vortexed thoroughly. A 10-μg meropenem disk was then added, and the suspension was incubated at 35°C in ambient air for 4 h. Meanwhile, a 0.5 McFarland suspension of *Escherichia coli* ATCC 25922 was prepared in sterile saline and used to inoculate a Mueller-Hinton agar plate according to the routine disk diffusion procedure within 15 min of suspension preparation. After the 4-h incubation, the meropenem disk was removed from the test suspension and placed onto the inoculated *E. coli* ATCC 25922 lawn. The plate was then incubated at 35°C in ambient air for 18–24 h. An inhibition zone diameter of 6–15 mm, or 16–18 mm with pinpoint colonies within the inhibition zone, was interpreted as mCIM positive. A zone diameter of ≥19 mm without pinpoint colonies was interpreted as negative. All other patterns were considered indeterminate and required repeat or additional testing.

**S1.1.2 EDTA-carbapenem inactivation method**

For eCIM, 20 μL of 0.5 mol/L EDTA was added to 2 mL of tryptic soy broth to obtain a final EDTA concentration of 5 mmol/L. The remaining procedure was performed in parallel with mCIM. A positive eCIM result was defined as an increase of ≥5 mm in the inhibition zone diameter relative to the paired mCIM result. The eCIM was interpreted only for isolates with a positive mCIM result.

**S1.2 Additional whole-genome sequencing and bioinformatics details**

Genomic DNA was extracted using the Tiangen Bacterial Genome DNA Extraction Kit according to the manufacturer’s instructions. Libraries were prepared using the NEBNext Ultra DNA Library Prep Kit according to the sequencing provider’s workflow. Illumina PE150 sequencing was performed by Shenzhen Haiyi Times Genomics. Quality-checked reads were assembled using SOAPdenovo v2.04, with gap closure using GapCloser v1.12. Kleborate v3.2.4 was run using its default allele-matching criteria. Acquired resistance genes were called using the default Kleborate thresholds of ≥90% nucleotide identity and ≥80% nucleotide coverage. Core genes were identified using Roary v3.12 with a BLASTP identity cutoff of 90%, and recombinant regions were removed using Gubbins v2.2.0. A maximum-likelihood tree was inferred using FastTree v2.1.7, with *Klebsiella oxytoca* ATCC 49131 as the outgroup.

**S1.3 Additional details of the in-house SYBR Green real-time PCR assay**

Primer pairs targeting *bla*_KPC_, *bla*_NDM_, *bla*_IMP_, *bla*_VIM_, and *bla*_OXA-48-like_ gene groups were developed in-house. The complete primer sequences and expected amplicon sizes are provided in Supplementary Table S1. Each target was tested in a separate singleplex SYBR Green real-time PCR reaction. For boiling extraction, bacterial colonies from overnight cultures were suspended in 200 μL of sterile nuclease-free water, heated at 100°C for 10 min, and centrifuged at 12,000 × g for 5 min. The supernatant was used as the DNA template. Each 20-μL reaction contained 10 μL of 2× SYBR Green Master Mix, forward and reverse primers each at a final concentration of 0.2 μM, 1 μL of DNA template, and nuclease-free water to the final volume. Amplification was performed on an ABI 7500 or SLAN-96 real-time PCR system using an initial denaturation step at 95°C for 10 min, followed by 40 cycles of 95°C for 15 s and 60°C for 60 s. Melting-curve analysis was performed after amplification.

Positive controls consisted of genotype-confirmed clinical isolates carrying the corresponding carbapenemase genes. Negative controls included a genotype-confirmed carbapenemase-negative *K. pneumoniae* isolate and a no-template control. Reactions with Ct values ≥35, nonspecific or multiple melting peaks, or melting temperatures inconsistent with the corresponding positive control were considered indeterminate and repeated using a freshly prepared DNA extract. If a positive control failed to amplify as expected or amplification occurred in the no-template control, the entire run was considered invalid and repeated. Final interpretation was based on the repeat result. Samples remaining indeterminate after repeat testing were reported as indeterminate.

**Supplementary Table S1. Primer sequences used for the in-house singleplex SYBR Green real-time PCR assays**

| **Target gene group** | **Forward primer sequence (5′–3′)** | **Reverse primer sequence**  **(5′–3′)** | ****Expected amplicon size (bp)**** |
| --- | --- | --- | --- |
| *bla*_KPC_ | TTACGGCAAAAATGCGCTGG | GGCGGCGTTATCACTGTATTG | 115 |
| *bla*_NDM_ | ACAAGATGGGCGGTATGGAC | AAGCTGGTTCGACAACGCAT | 74 |
| *bla*_IMP_ | TGGCCAAAGTCCGCTAAA | CCGCCTGCTCTAATGTAAGT | 115 |
| *bla*_VIM_ | CGACTTTACCAGATTGCCGA | CCCCACGCTGTATCAATCAA | 131 |
| *bla*_OXA-48-like_ | AGCATTTTTACCCGCATCTACCTT | CCACTTAAAGACTTAAAGACTTGGTGTTCATCCTT | 102* |

All primer sequences are presented in the 5′–3′ orientation. Each primer pair was used in a separate singleplex SYBR Green reaction.

*The expected 102-bp amplicon for *bla*OXA-48-like includes the 10-nt 5′ non-templated extension of the reverse primer; the corresponding template-derived region is 92 bp.

**S1.4 Additional details of the low-inoculum spread-plate colony-recovery assay**

**S1.4.1 Plate preparation, inoculation, and colony counting**

Mueller-Hinton agar plates containing imipenem or meropenem were prepared at final concentrations of 1, 2, and 4 mg/L. Antibiotic-free plates were prepared in parallel. The 10,000-fold bacterial dilution was generated by four successive 10-fold dilutions in sterile saline. A 50-μL aliquot was applied to each 9-cm plate and spread evenly over the entire agar surface using a sterile swab.

The inoculum had been optimized in preliminary experiments to yield 50–300 CFU on antibiotic-free control plates. Plates were incubated at 35°C in ambient air for 18–24 h and colonies were counted manually. Plates showing contamination, confluent or otherwise uninterpretable growth, or an antibiotic-free control count outside the predefined countable range were repeated.

**S1.4.2 Relative recovery and variability assessment**

For each isolate, antibiotic, and concentration, relative recovery was calculated using the paired colony-count ratio defined in the main Methods. Relative-recovery values were expressed as decimals for HDI calculation and as percentages for visualization in Figure 4. Values slightly above 100% were retained without adjustment because minor variation may arise from suspension homogeneity, spreading, colony distribution, and manual counting.

Following the initial concentration-series screening experiment, repeatability of the 1-mg/L colony-recovery assay was assessed in three independent repeat experiments performed on separate days using freshly prepared bacterial suspensions. Duplicate plates were included for the antibiotic-free control, IPM 1 mg/L, and MEM 1 mg/L conditions for each isolate in each experiment. Intra-assay variability was assessed using the relative percent difference (RPD) between duplicate colony counts, calculated as RPD (%) = |N₁ − N₂| / [(N₁ + N₂)/2] × 100, where N₁ and N₂ represent the colony counts from the two duplicate plates. When both duplicate counts were zero, RPD was assigned a value of zero. Duplicate colony counts were averaged before calculation of experiment-specific relative-recovery and HDI values. Between-experiment variability across the three repeat experiments was summarized using the mean, standard deviation (SD), and range of experiment-specific HDI values. Rank consistency across experiments was further evaluated by pairwise Spearman rank correlations. Agreement between the initial screening result and the repeat experiments was explored by comparing the initial HDI with the mean HDI across the three repeat experiments using Spearman rank correlation, the absolute and paired differences, and the Wilcoxon signed-rank test. Isolate-level replicate colony counts, relative-recovery values, HDI calculations, RPD values, and repeatability metrics are provided in Table S2.
